# Multimarker Approach to Identify Patients with Coronary Artery Disease at High Risk for Subsequent Cardiac Adverse Events: The Multi-Biomarker Study

**DOI:** 10.3390/biom10060909

**Published:** 2020-06-15

**Authors:** Georgiana-Aura Giurgea, Katrin Zlabinger, Alfred Gugerell, Dominika Lukovic, Bonni Syeda, Ljubica Mandic, Noemi Pavo, Julia Mester-Tonczar, Denise Traxler-Weidenauer, Andreas Spannbauer, Nina Kastner, Claudia Müller, Anahit Anvari, Jutta Bergler-Klein, Mariann Gyöngyösi

**Affiliations:** 1 Department of Angiology, Internal Medicine II, Medical University of Vienna, 1090 Vienna, Austria; georgiana-aura.giurgea@meduniwien.ac.at; 2 Department of Cardiology, Internal Medicine II, Medical University of Vienna, 1090 Vienna, Austria; katrin.zlabinger@meduniwien.ac.at (K.Z.); alfred.gugerell@meduniwien.ac.at (A.G.); dominika.lukovic@meduniwien.ac.at (D.L.); b.syeda@internist-nord.at (B.S.); ljubica.mandic@gmail.com (L.M.); noemi.pavo@meduniwien.ac.at (N.P.); julia.mester-tonczar@meduniwien.ac.at (J.M.-T.); denise.traxler-weidenauer@meduniwien.ac.at (D.T.-W.); andreas.spannbauer@meduniwien.ac.at (A.S.); nina.kastner@meduniwien.ac.at (N.K.); claudia.mueller@meduniwien.ac.at (C.M.); anahit.anvari@meduniwien.ac.at (A.A.); jutta.bergler-klein@meduniwien.ac.at (J.B.-K.)

**Keywords:** multimarker approach, adverse event, risk prediction, canonical discriminant analysis, C-statistics, coronary artery disease

## Abstract

In our prospective non-randomized, single-center cohort study (*n* = 161), we have evaluated a multimarker approach including S100 calcium binding protein A12 (S100A1), interleukin 1 like-receptor-4 (IL1R4), adrenomedullin, copeptin, neutrophil gelatinase-associated lipocalin (NGAL), soluble urokinase plasminogen activator receptor (suPAR), and ischemia modified albumin (IMA) in prediction of subsequent cardiac adverse events (AE) during 1-year follow-up in patients with coronary artery disease. The primary endpoint was to assess the combined discriminatory predictive value of the selected 7 biomarkers in prediction of AE (myocardial infarction, coronary revascularization, death, stroke, and hospitalization) by canonical discriminant function analysis. The main secondary endpoints were the levels of the 7 biomarkers in the groups with/without AE; comparison of the calculated discriminant score of the biomarkers with traditional logistic regression and C-statistics. The canonical correlation coefficient was 0.642, with a Wilk’s lambda value of 0.78 and *p* < 0.001. By using the calculated discriminant equation with the weighted mean discriminant score (centroid), the sensitivity and specificity of our model were 79.4% and 74.3% in prediction of AE. These values were higher than that of the calculated C-statistics if traditional risk factors with/without biomarkers were used for AE prediction. In conclusion, canonical discriminant analysis of the multimarker approach is able to define the risk threshold at the individual patient level for personalized medicine.

## 1. Introduction

Cardiovascular mortality or morbidity risk scores are essential in primary and secondary prevention of cardiovascular adverse events (AE), and are mostly based on traditional cardiovascular risk factors, such as hypertension, diabetes mellitus, hyperlipidemia, smoking, and family history. Risk scores are created and calculated for patients with acute coronary syndrome: Thrombolysis in Myocardial Infarction (TIMI), Platelet Glycoprotein IIb/IIIa in Unstable Angina: Receptor Suppression Using Integrilin (PURSUIT), the Global Registry of Acute Coronary Events (GRACE) [1], for stable angina pectoris: Framingham Risk Score [2], Vienna and Ludwigshafen Coronary Artery Disease (VILCAD) score [3], or for general patient population, such as the SCORE-European High Risk Chart [4]. Previous studies suggested, that biomarkers of inflammation, fibrinolysis or fibrin formation, endothelial function, oxidative stress, or renal or heart function parameter enhance the value of risk prediction and enable early intervention and prevention of subsequent cardiovascular adverse events (AE) [5]. Adding of routinely used biomarkers, such as troponin T or NT-pro-brain natriuretic peptide (pro-BNP) or blood cholesterol level to the traditional risk factors enhanced moderately the risk prediction in the individual patients [6]. Single or combined new biomarkers, such as copeptin, neutrophil gelatinase-associated lipocalin (NGAL), or soluble urokinase plasminogen activator receptor (suPAR) have been tested and validated in larger cohorts of patients, anticipating prognostic values of these biomarkers [7,8,9]. However, statistical association between biomarkers and onset of AEs in a large patient cohort is not necessarily useful for personalized risk stratification [10]. In contrast with single blood marker analysis, the multi-biomarker approach by using traditional or new circulating proteins might have the potential to enhance the risk stratification in individual patients.

We have selected 7 non-traditional biomarkers of different classes, possessing diagnostic or prognostic significance in forecasting of cardiovascular AEs, and combined them in a multi-biomarker approach to yield prognostic value for a single patient. The selected biomarkers were as follows: S100 calcium binding protein A12 (S100A1) and interleukin 1 like-receptor-4 (ST2, IL1R4) (markers of inflammation), adrenomedullin and copeptin (vasoactive markers), NGAL (tissue injury marker), suPAR (fibrinolysis marker), and ischemia modified albumin (IMA) (oxidative stress marker).

S100A12 protein is released from activated macrophages and has been proposed to contribute to the acceleration of atherosclerosis [11,12]. Midregional pro-adrenomedullin (MR-proADM, adrenomedullin) is a potent vasodilatatory peptide, and a marker of hemodynamic stress [13,14]. Copeptin, the C-terminal portion of the vasopressin prohormone, is released stoichiometrically with vasopressin in the neurohypophysis, and in combination with troponin T, improved the early risk stratification of patients presenting with acute chest pain [15]. NGAL is a marker of acute kidney injury but has also been associated with different cardiovascular diseases and elevated in patients with heart failure after myocardial infarction [16]. The suPAR is a plasma marker of low-grade inflammation that has been associated with cardiovascular risk [17,18]. IL1R4 is up-regulated in conditions with increased myocardial strain [19,20,21,22,23]. IMA is a sensitive biomarker of myocardial ischemia after percutaneous coronary intervention (PCI), or during coronary artery bypass surgery (CABG), and used for risk stratification tool for suspected acute coronary syndrome or as a prognostic marker in patients with cardiopulmonary resuscitation [24,25]. All of these biomarkers have been associated with cardiovascular events in patients with coronary artery disease (CAD).

The aim of the multi-biomarker study was to evaluate the multimarker approach for personalized medicine including the selected biomarkers S100A1, adrenomedullin, copeptin, NGAL, suPAR, IL1R4, and IMA in prediction of subsequent cardiac AEs during 1-year follow-up (FUP) in patients with CAD.

## 2. Materials and Methods

### 2.1. Study Design

The multi-biomarker study and biobank is a prospective non-randomized, single-center cohort study, including patients with either stable CAD or subacute myocardial infarction (AMI)—including ST-segment elevation myocardial infarction (STEMI) or non-STEMI (NSTEMI). Blood samples were taken to assess the discriminatory values of the single or multi-biomarker approach for prediction of major cardiac AE during the 1-year FUP period. The study was conducted at the Department of Cardiology, Medical University of Vienna in accordance with the Declaration of Helsinki (1975) and approved by the Ethics Committee of the Medical University of Vienna (EK Nr: 2011/1091 and 1276/2019). Written informed consent was obtained from all patients.

### 2.2. Patient Population, Inclusion and Exclusion Criteria

Patients with stable CAD and recent AMI were included. Stable CAD was defined as previously angiographical documented CAD, with/without previous PCI, AMI, CABG, with no angina pectoris or inducible myocardial ischemia at the time of the study inclusion, recruited in the outpatient clinic. Recent AMI was defined as current STEMI or NSTEMI after primary PCI, recruited in the internal ward before hospital discharge post-AMI. The mean time of blood collection in the AMI group was 2.4 ± 0.3 days post AMI-onset.

Inclusion criteria were proven CAD (stable CAD or recent AMI) in patients older than 19 years, and willing to participate in the study.

Exclusion criteria were known cancer, acute or chronic infective or auto-immune inflammatory diseases, hemodynamically significant valvular diseases, hypertrophic or restrictive cardiomyopathy, congenital heart disease, previous acute renal failure, major surgery within the last 3 months, liver diseases requiring treatment, inability or unwillingness to comply with the study protocol, and chronic renal failure with glomerular filtration rate (GFR) ≤ 40.

### 2.3. Endpoints of the Study

The primary endpoint of the study was to assess the combined discriminatory predictive value of the biomarkers S100A1, adrenomedullin, copeptin, NGAL, suPAR, IL1R4, and IMA in prediction of cardiac AE, defined as recurring myocardial infarction, coronary revascularization by PCI or CABG, death, stroke, hospitalization due to angina pectoris without revascularization or heart failure and implantation of pacemaker or automatic implantable cardioverter defibrillator /AICD/ due to malignant arrhythmias, in comparison with the usual logistic regression or C-statistics.

The secondary endpoints of the study were the levels of the 7 selected biomarkers in the subgroups of stable CAD (group CAD) and recent AMI (group AMI), association between the new biomarkers and NT-proBNP.

### 2.4. Clinical Data Collection

Baseline medical history (age, gender, presence of atherosclerotic risk factors, such as hypertension, diabetes mellitus, hyperlipidemia, smoking, previous AMI, PCI, CABG, peripheral and/or carotid artery disease), current medical treatment (daily regular dose of aspirin, clopidogrel, beta-blocker, ACE inhibitor or ARB), and transthoracic echocardiography (TTE) data (global left ventricular function expressed as wall motion score index) were recorded at the study inclusion and at the 1-year control clinical investigation.

At 1-year FUP, patients were invited to medical examination and TTE. The follow-up clinical history was documented, including the AEs.

### 2.5. Laboratory Procedures

Fasting venous blood samples were obtained into commercially available tubes. Serum and plasma aliquots were stored at −80 °C until biomarker assay. Troponin T, N-terminal-proBNP (NT-proBNP), and creatine kinase MB fraction (CK-MB) were assessed using a routine diagnostic analyzer in the hospital laboratory.

Biomarker concentrations in samples were measured by commercially available enzyme-linked immunosorbent assays (ELISA): S100A12 and copeptin from Cloud-Clone Corp. (Houston, TX, USA); suPAR, adrenomedullin and IMA from MyBioSource Inc. (San Diego, CA, USA); NGAL (Lipocalin 2) from ABCAM (Cambridge, UK) and IL1R4/ST2 from Sigma-Aldrich Co (St. Louis, MO, USA). All assays were performed according to the manufacturer’s instructions.

### 2.6. Statistics

The statistical analysis was performed using SPSS^®^ version 23 (SPSS Inc, Chicago, IL). Continuous parameters with normal or non-normal distribution were expressed as mean ± standard deviations or median with interquartile range, respectively. Categorical variables were listed as number and percentages. For all tests, two-sided analyses were used and the significance level was set at *p* < 0.05.

The predictive power of the biomarkers in classification of AEs was calculated by using canonical discriminant function analysis. The predictor (independent) variables were the 7 selected biomarkers and NT-proBNP. Wilk’s lambda test was used to test the significance between the groups with/without AEs. Parameters with discriminatory function <0.3 (non-significant correlation with the other parameter with low discriminatory function between the groups) were stepwise excluded from the further discriminant function model. A discriminant score was calculated by using weighted combination of the biomarkers with >3 discriminatory function. The means and SDs of the discriminant scores and the weighted mean score cut-off values between the groups (event yes/no) were calculated and described as centroid. Sensitivity and specificity of the discriminatory score were determined from the classification results. To confirm the correctness of the scoring, individual patient discriminant scores were calculated, and the mean scores of the groups (AE or non-AE) were compared by 2-sided Student *t*-test.

Multivariate binary logistic regression analysis was performed to analyze the traditional risk factors, supplemented with the biomarkers predictive for subsequent cardiac AE. Continuous parameters were transformed to binary parameter using the quadratic difference between supposed predictive value (75% interquartile value) and observed binary outcome (0 for no event, 1 for event) for each patient. The following parameters were included into the analysis: male gender, age, presence of atherosclerotic risk factors (diabetes mellitus, hypertension, smoking, hyperlipidemia), all 7 selected biomarkers, and NT-proBNP. C-statistics was performed to predict risk and sensitivity and specificity of all included clinical and biomarker risk factors.

Linear regression analysis was used to search association between NT-proBNP (as established prognostic marker) and the 7 selected biomarkers: S100A1, adrenomedullin, copeptin, NGAL, suPAR, IL1R4, and IMA.

## 3. Results

A total of 181 patients were included in the study. Due to comorbidities possibly or definitively influencing the blood levels of the selected 7 biomarkers, such as moderate to severe acute or chronic renal failure, or malignant or other chronic disease diagnosed after study inclusion, 20 patients were excluded from the biomarker measurement study. Therefore, blood levels of the 7 selected biomarkers were measured in 161 patients and these 161 patients were included in the further analyses.

### 3.1. Clinical Events during the 1-Year FUP

In total, 48 cardiac AEs occurred. Four patients died, 8 patients experienced re-AMI, and 23 patients were hospitalized for heart failure or angina pectoris, or implantation of pacemaker or automatic implantable cardioverter defibrillator. Coronary revascularization was performed in 13 patients.

Table 1 lists the patient characteristics, and the baseline blood levels of biomarkers in all patients, and the groups AE and non-AE. Patients with adverse event at the follow-up had more often diabetes mellitus and previous bypass surgery, and more patients were smokers. NGAL, suPAR, and IL1R4 were significantly higher in group AE, however, large scatter of the data was observed.

### 3.2. Multimarker Approach for Prediction of Adverse Events

The structure matrix of the canonical discriminant analysis including all selected 7 biomarkers showed low (<0.3) Pearson correlation between S100A1, adrenomedullin, copeptin, and IMA and the other biomarkers, with a low sensitivity of 43.5% and high specificity of 94.5% in prediction of AE. In order to increase the sensitivity of the multiple biomarker testing, we have stepwise excluded biomarkers with low level of correlation between the other markers. Finally, including the factors with the strongest discriminant predictive power, NGAL, suPAR, and IL1R4, the canonical correlation coefficient was 0.496, with a Wilk’s lambda value of 0.001, resulting in a discriminant model of:

Discriminant score = −2.01 + 0.025 × NGAL_i_ + 0.130 × suPAR_i_ + 0.001 × IL1R4_i_,

where i: individual value of the same patient.

**Figure 1:**
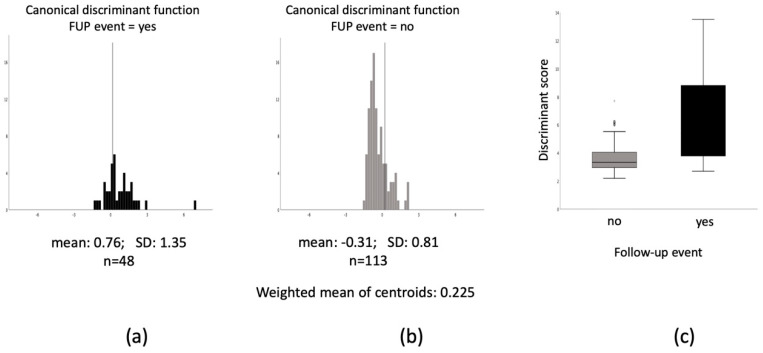
Discriminant scores, weighted mean of centroids, and C-statistics of patients experiencing cardiovascular adverse events. (**a**) and (**b**) discriminant score values of patients with (**a**) or without (**b**) adverse events at the 1-year follow-up (FUP); (**c**) mean (SD) discriminant scores of patients with or without adverse events at the FUP.

The weighted mean discriminant score (cutting point, centroid) was 0.225 (Figure 1), resulting in a sensitivity of 79.4% and specificity of 74.3% in prediction of adverse event, if the calculated discriminant equation was used. Accordingly, 76.9% of patients have been correctly classified to groups AE or non-AE if the calculated scores were used in the individual patient level. Patients with AEs had significantly higher discriminant value (calculated from the discriminant equation) as compared with patients without events (Figure 1).

**Figure 2:**
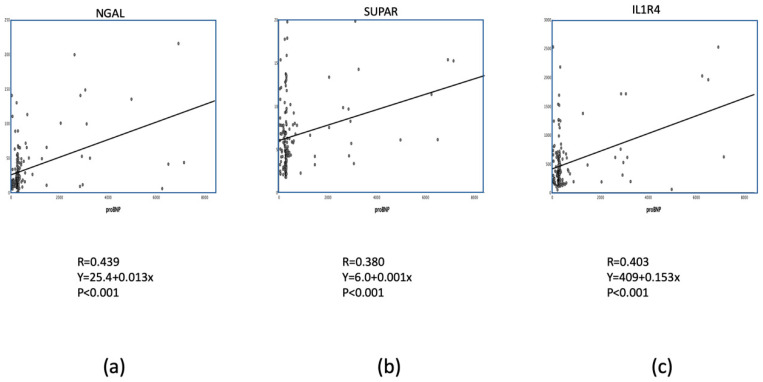
Significant correlation between N-terminal-pro-brain natriuretic peptide (NT-proBNP) (pg/mL) and new biomarkers with (**a**) neutrophil gelatinase-associated lipocalin (NGAL) (pg/mL); (**b**) soluble urokinase plasminogen activator receptor (SUPAR) (ng/mL); (**c**) interleukin 1 like-receptor-4 (ST2, IL1R4) (pg/mL).

Interestingly, including NT-proBNP into the model did not enhance the power of the analysis, e.g., it did not increase the sensitivity/specificity values of the event prediction, most probably due to the significant association between NT-proBNP and NGAL or suPAR or IL1R4, respectively (Figure 2).

### 3.3. Risk Prediction Including Clinical Variables by Using Logistic Regression and C-Statistics

**Figure 3:**
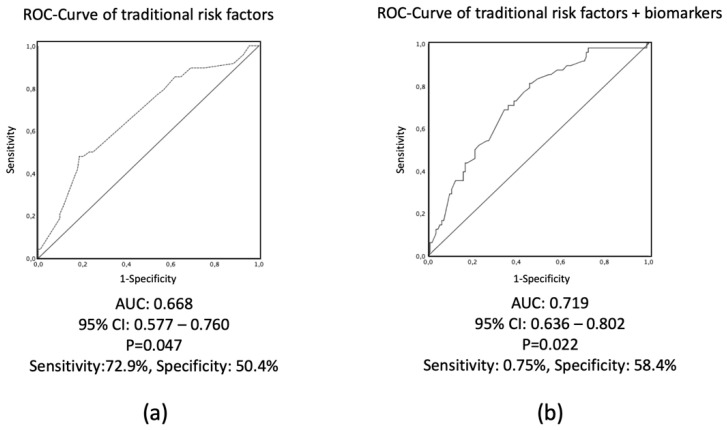
C-statistics and receiver operator characteristics (ROC) curves of predictive adverse events of patients experiencing cardiovascular adverse events. (**a**) ROC curve analysis if traditional risk factors (male gender, older age, diabetes, hypertension, hyperlipidemia, and smoking) were included into the analysis; (**b**) ROC curve if 7 biomarkers and NT-proBNP were additionally included into the analysis of traditional risk factors.

Binary logistic regression analysis including the traditional risk factors (age, male gender, diabetes, hypertension, hyperlipidemia, and smoking) could not reveal any significant predictors for adverse events. C-statistics revealed a C value (area under the curve of ROC analysis) of 0.58, with no significant prediction value for AEs (Figure 3).

Entering of the selected 7 biomarkers and NT-proBNP into the logistic regression analysis containing also the traditional risk factors did not result in any significant predictors. However, adding the 8 biomarkers increased moderately but significantly the prediction of AEs by a C value of 0.668 (*p* = 0.047) (Figure 3) in the C-statistics, with an optimized sensitivity and specificity values of 72.9% and 50.4%.

### 3.4. Diagnostic Value of Selected Biomarkers in Recent AMI

Patients were classified into subgroups CAD (*n* = 39) and AMI (*n* = 42). Table 2 lists the baseline clinical variables of the subgroups. Patients in the group CAD had a higher incidence of previous AMI and PCI. Patients in the AMI group had higher NT-proBNP, CK, and TnT 2.4 ± 0.3 days post AMI onset.

**Figure 4:**
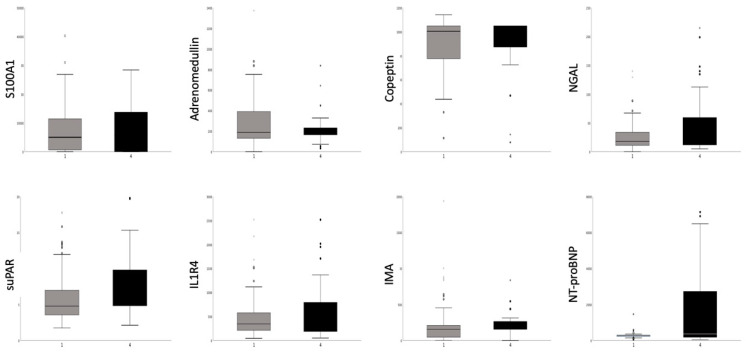
Blood levels of biomarkers of patients with stable coronary artery disease (subgroup CAD) or with recent acute myocardial infarction (subgroup AMI). S100 calcium binding protein A12 (S100A1) (ng/mL), adrenomedullin (ng/mL), copeptin (pg/mL), neutrophil gelatinase-associated lipocalin (NGAL) (pg/mL), soluble urokinase plasminogen activator receptor (suPAR) (ng/mL), interleukin 1 like-receptor-4 (ST2, IL1R4) (pg/mL), ischemia modified albumin (IMA) ng/mL), N-terminal-pro-brain natriuretic peptide (NT-proBNP) (pg/mL). No significant differences between the subgroups.

Table 2 and Figure 4 shows the blood levels of biomarkers. Beside the significantly elevated NT-proBNP value in the AMI group, from the 7 new biomarkers, only suPAR showed a trend towards higher value in the AMI group.

## 4. Discussion

To our knowledge, this is the first study to use the multi-biomarker approach by using the absolute values of the blood biomarkers, by using canonical discriminant analysis. Our study demonstrated, that (1) canonical discriminant analysis of the multimarker approach is able to define risk threshold in the individual single patient level; (2) the weighted mean discriminant score (cutting point, centroid) resulted in a sensitivity of 79.4% and specificity of 74.3% in prediction of adverse event, if calculated discriminant equation was used, with 76.9% of patients classified correctly to groups AE or non-AE; (3) classical C-statistics for adverse event prediction including the traditional risk factors (age, male gender, diabetes, hypertension, hyperlipidemia, and smoking) and 8 biomarkers revealed a C value of 0.719 (*p* = 0.022), with a sensitivity and specificity values of 75.0% and 58.4%; (4) canonical discriminant analysis using the absolute values of biomarkers of coronary artery disease has a better sensitivity and specificity in prediction of adverse events than the usual logistic regression or C-statistics in a single patient level. Additionally, our study revealed low diagnostic value of the selected 7 biomarkers in patients with recent AMI.

We have selected different class biomarkers (inflammation, vasoactive, fibrinolysis, oxidative stress, and tissue injury), since the biomarker of the same or similar classes may have coincidental information, failing to give additive values in event prediction. Additionally, a limited number of non-correlative markers may better improve the risk stratification than a larger number of biomarkers acting in the similar biological pathway. Most probably, this might be the reason, why the additive biomarker NT-proBNP did not improve the sensitivity/specificity values in our study, since NT-proBNP showed a significant correlation with all 3 biomarkers entered into the discriminant equation.

Previous studies investigating the role of multiple biomarkers in patients with or without CAD have demonstrated only modest improvement in predictive accuracy, by using C-statistic, if biomarkers were added to traditional clinical risk factors [26,27]. C-statistics are commonly applied to quantify and evaluate the risk score in patients with predefined AEs [28]. In C-statistics, a receiver operating characteristic (ROC) curve displays the diagnostic capacity of a binary classification system. In contrast discriminant analysis, it is allowed to utilize the continuous absolute values of the measured parameters without compulsory transformation of the continuous variables to categorical ones. Therefore, this analysis type may separate the groups by the most exact way and discard parameters mathematically which have less prediction value in the group classification. We did not include the established clinical risk factors into our discriminant model, because (1) dichotomy variables might weaken the prediction of AEs in our mathematical model; (2) the control of the cardiovascular risk factors via first and second prevention and guided therapies modify the course of heart diseases, effectively balancing the pre-existing risk; and (3) presence of multiple risk factors influences the co-incident risk factors, such as diabetic nephropathy results in hypertension, or male patients have higher risk for event in younger age than woman in similar age.

The sensitivity and specificity values of our discriminant model were 79.4.% and 74.3%, below the expected prediction values of over 80%, even if Wilk’s lambda statistic value was significant. This is probably due to the heterogeneous patient population. We recognized a large scatter of the blood levels of the individual biomarkers, raising methodological concerns of the available ELISA kits.

The usual multivariate binary logistic regression model including the traditional risk factors did not reveal any significant predictors, even not, if the biomarkers (7 selected and NT-proBNP) were included into the model with mandatory transformation of the continuous variables to categorical ones. In contrast, C-statistics presented non-significant predictor values of the traditional risk factors, but adding the 8 biomarkers to the model improved the prediction significantly, albeit moderately. However, this latter approach still presented a less predictive accuracy with less optimal sensitivity and specificity values. The discrepancies between the outcomes of the logistic regression and C-statistics indicate the inconsistencies in statistical results, if individual patient risk prediction is required.

Our study has some limitations. There are several biomarkers associated with autoimmune diseases (such as copeptin with multiple sclerosis, NGAL, and systemic lupus erythematosus), therefore we have excluded patients with autoimmune disorders from our study [29,30]. We have also excluded patients with moderate or severe renal failure, because circulating levels of several biomarkers (copeptin, NT-proBNP, NGAL) are influenced by chronic kidney disease, independent from the coronary artery disease. Most of the assumptions needed for the correct calculation of the discriminant analysis were fulfilled, e.g., independent cases, within-group variance-co-variance matrices are equal across the groups; categorical parameter is used for group variable. However, some predictor variables do not have multivariate normal distribution, but our analysis including 161 patients is robust enough to overcome this assumption, and has high sensitivity and specificity to balance the outliers. As usual, in academic studies, our patient collective is heterogeneous, with a relatively small effect size in terms of AEs. However, a multimarker approach with calculated discriminant scores might be helpful for individual patient stratification, and improves the risk calculation at the individual patient level. Measuring more biomarkers would definitively increase in cost, with eventually only minor additive value in risk prediction.

## 5. Conclusions

Our canonical discriminatory model with multimarker approach is able to define a risk threshold at the individual patient level, additive to the conventional risk stratifications as part of personalized medicine in cardiology.

## Figures and Tables

**Table 1:** Clinical and laboratory parameters of patients with coronary artery disease with/without cardiac adverse events (AE) during the 1-year follow-up.

Clinical and Laboratory Parameter	All Patients *n* = 161	Group AE *n* = 48	Group Non-AE *n* = 113	*p* Value between Groups
Male gender n (%)	126 (78.3%)	35 (72.9%)	91 (80.5%)	0.301
Age (y; mean ± SD)	67.0 ± 11.9	70.4 ± 11.1	65.6 ± 12.0	0.017
Diabetes mellitus n (%)	41 (25.5%)	18 (37.5%)	23 (20.4%)	0.030
Hypertension n (%)	129 (80.1%)	41 (85.4%)	88 (77.9%)	0.388
Hyperlipidemia n (%)	117 (72.7%)	37 (77.1%)	80 (70.8%)	0.447
Smoking n (%)	58 (36.0%)	26 (55.3%)	32 (28.3%)	0.011
Previous MI n (%)	83 (51.6%)	29 (60.4%)	54 (47.8%)	0.295
Previous PCI n (%)	85 (52.8%)	22 (45.8%)	63 (55.8%)	0.301
Previous CABG n (%)	31 (19.3%)	17 (35.4%)	14 (12.4%)	0.002
PAD n (%)	20 (12.4%)	9 (18.8%)	11 (9.7%)	0.123
Carotid artery disease n (%)	28 (17.4%)	9 (18.8%)	19 (17.0%)	0.822
Aspirin (%)	161 (100%)	48 (100%)	113 (100%)	1
Clopidogrel n (%)	132 (82.0%)	38 (79.2%)	94 (83.2%)	0.408
Beta-blocker n (%)	152 (94.4%)	43 (89.6%)	109 (96.4%)	0.398
ACE-inhibitor/ARB n (%)	146 (90.7%)	42 (87.5%)	104 (92.0%)	0.780
NT-proBNP (median; Q) (pg/mL)	280 (232; 335)	282 (243; 507)	279 (232; 309)	0.224
Troponin T (ng/L) (median; Q) (ng/mL)	0.01 (0.01; 0.41)	0.01 (0.01; 0.04)	0.01 (0.01; 0.08)	0.313
Creatine kinase (U/L) (median; Q)	116 (66; 188)	88 (67; 177)	118 (66; 106)	0.541
Baseline WMSI (mean ± SD)	1.31 ± 0.46	1.22 ± 0.45	1.34 ± 0.47	0.115
Follow-up WMSI (mean ± SD)	1.31 ± 0.51	1.28 ± 0.51	1.32 ± 0.51	0.651
**Selected biomarkers (Median, 25% and 75% Quartiles)**				
S100A1(ng/mL)	5504 (0; 11858)	5311 (0; 14462)	5674 (0; 11327)	0.724
Adrenomedullin (ng/mL)	189 (146; 328)	178 (144; 279)	194 (150; 343)	0.187
Copeptin (pg/mL)	1049 (817; 1049)	938 (731; 1049)	1049 (844; 1049)	0.058
NGAL (pg/mL)	18.5 (11.0; 39.0)	40.3 (16.5; 57.7)	16.2 (9.9; 27.6)	<0.05
suPAR (ng/mL)	5.37 (3.70; 8.51)	9.18 (3.58; 11.99)	4.94 (3.70; 6.95)	<0.05
IL1R4 (pg/mL)	347 (211; 616)	542 (222; 769)	318 (193; 548)	<0.05
IMA (ng/mL)	167 (53; 219)	156 (34; 211)	178 (76; 221)	0.120

MI: myocardial infarction; PCI: percutaneous coronary intervention; CABG: coronary artery bypass graft surgery; PAD: peripheral artery disease; ACE: angiotensin converting enzyme; ARB: angiotensin receptor blocker; NT-proBNP: N-terminal pro-brain natriuretic peptide; WMSI: wall motion score index, S100A1: S100 calcium binding protein A12; NGAL: neutrophil gelatinase-associated lipocalin; suPAR: soluble urokinase plasminogen activator receptor; IL1R4: interleukin 1 like-receptor-4; IMA: ischemia modified albumin. Q: 25% and 75% quartiles.

**Table 2:** Clinical and laboratory parameters of subgroup of patients with stable coronary artery disease (subgroup CAD) or with recent acute myocardial infarction (subgroup AMI).

Clinical and Laboratory Parameter	Subgroup CAD *n* = 111	Subgroup AMI *n* = 50	*p* Value between the Groups
Male gender n (%)	89 (80.2%)	37 (74.0%)	0.412
Age (y; mean ± SD)	68.4 ± 11.5	64.0 ± 12.3	0.036
Diabetes mellitus n (%)	34 (30.6%)	7 (14.0%)	0.031
Hypertension n (%)	92 (82.9%)	37 (74.0%)	0.205
Hyperlipidemia n (%)	77 (69.4%)	40 (80.0%)	0.185
Smoking n (%)	34 (30.6%)	24 (48.0%)	0.167
Previous MI n (%)	71 (64.0%)	12 (24.0%)	<0.001
Previous PCI n (%)	74 (66.7%)	11 (22.0%)	<0.001
Previous CABG n (%)	25 (22.5%)	6 (12.0%)	0.135
PAD n (%)	15 (13.5%)	5 (10.0%)	0.614
Carotid artery disease n (%)	23 (20.7%)	5 (10%)	0.117
Aspirin n (%)	111 (100%)	50 (100%)	1
Clopidogrel n (%)	92 (82.9%)	40 (80.0%)	0.666
Beta-blocker n (%)	102 (91.9%)	50 (100%)	0.863
ACE-inhibitor/ARB n (%)	102 (91.9%)	44 (88.0%)	0.814
NT-proBNP (median; Q) (pg/mL)	278 (241; 301)	359 (182; 881)	0.029
Troponin T (ng/L) (median; Q) (ng/mL)	0.01 (0.01; 0.01)	0.26 (0.04; 2.33)	<0.001
Creatine kinase (U/L) (median; Q)	85 (55; 137)	248 (131; 569)	<0.001
Baseline WMSI (mean ± SD)	1.29 ± 0.43	1.34 ± 0.54	0.611
Follow-up WMSI (mean ± SD)	1.29 ± 0.46	1.34 ± 0.64	0.716
FUP events n (%)	30 (27.0%)	18 (36.0%)	0.268
Hospitalization n (%)	16 (14.4%)	7 (14.0%)	
Acute MI n (%)	6 (5.4%)	2 (4.0%)	
Revascularization n (%)	6 (5.4%)	7 (14%)	
Death n (%)	2 (1.8%)	2 (4.0%)	

MI: myocardial infarction; PCI: percutaneous coronary intervention; ACBP: aorto-coronary bypass surgery; PAD: peripheral artery disease; ACE: angiotensin converting enzyme; ARB: angiotensin receptor blocker; NT-proBNP: N-terminal pro-brain natriuretic peptide; WMSI: wall motion score index; Q: 25% and 75% quartiles.
